# Use of Measuring Tools in Practice Development Projects: A Critical Perspective

**DOI:** 10.1177/0008417421994365

**Published:** 2021-03-08

**Authors:** Stinne Glasdam, Tobba Therkildsen Sudmann

**Keywords:** Empowerment, Foucault, Governmentality, Older adults, Reablement projects, Pouvoir d'agir, Foucault, Gouvernementalité, Adultes plus âgés, Projets de services ambulatoires à domicile

## Abstract

**Background.:**

Occupational therapists are generally positive towards use of measuring tools. However, such use may be problematic.

**Purpose.:**

To illuminate hidden and adverse effects of using measuring tools in occupational therapy.

**Method.:**

A Foucauldian inspired thematic analysis of the use of measuring tools in 13 reports of practice development projects in Denmark.

**Findings.:**

Three themes were constructed: “Categorisation of loss”, “Conduct of conduct: Self-tracking and competition”, and “Conforming to expected forms of everyday living”. Measuring tools tended to produce generalised truths about older adults and were used to predict outcome of or access to reablement programs. The measurements guided both older people and professionals, and measurements created both motivation and resistance. The tools served as an extension of the healthcare professionals’ authority.

**Implications.:**

When appropriately situated, measuring tools have the potential to empower and enhance older adults’ lives and should be the focus of greater clinical attention.

## Abstract (fr)

**Description.:**

Les ergothérapeutes sont généralement en faveur des outils d’évaluation. Toutefois, l’utilisation de ces outils pourrait poser certains problèmes.

**But.:**

Faire la lumière sur les effets cachés ou néfastes associés à l’utilisation d’outils d’évaluation en ergothérapie.

**Méthodologie.:**

Analyse thématique, selon l’approche foucaldienne, de l’utilisation d’outils d’évaluation dans 13 rapports de projets de développement de la pratique au Danemark.

**Résultats.:**

Trois thèmes ont été construits : “catégorisation de la perte”, “conduite de la conduite : suivi personnel et compétition” et “conformité aux modes de vie attendus”. Dans l’ensemble, les outils d’évaluation ont produit des vérités générales au sujet des adultes d’un certain âge, et ils ont été utilisés pour prédire les résultats de programmes de revalidation ou pour y avoir accès. Les évaluations ont guidé aussi bien les personnes d’un certain âge que les professionnels, et ont engendré à la fois motivation et résistance. Les outils d’évaluation ont permis une extension des pouvoirs des professionnels de la santé.

**Implications.:**

Quand les outils d’évaluation sont employés dans des situations pertinentes, ils ont le potentiel d’apporter des changements positifs dans la vie des adultes plus âgés et de leur permettre de se prendre en charge; par conséquent, ces outils devraient faire l’objet d’une plus grande attention clinique.

## Introduction

This article focuses on the use of measuring tools in time-limited practice development projects in homecare services for older adults in Danish municipalities during the past decade. These projects offered short-term intensive home-based inter-disciplinary assessments, treatments, and activities—often referred to as reablement programs. Reablement builds on the premise that age does not impede learning, improvement of physical capacity or social engagement, and/or re-learning of the day-to-day skills needed to encourage self-confidence and self-management, support independence, and promote healthy living among older adults (Aspinal et al. 2016). To be able to identify people eligible for home-based reablement services, older adults’ needs, wants, and capacities have to be assessed and documented. Currently, measuring tools are gaining momentum in healthcare, and in professionals’ encounters with potential recipients of home-based services (de Vet et al., 2011). The different assessments, tests, and tools provide documentation of needs and capacities, that is, the potential for improving function. Measurement outcomes are then used to justify priority settings and allocation of scarce public resources. At the same time, measurements and associated procedures are social practices that effect the older adults and the occupational therapists beyond the golden standards of measurements (de Vet et al, 2011).

The aim of this article is to illuminate hidden and adverse effects of using measuring tools in occupational therapy. A Foucauldian lens is used analytically to illuminate what measuring tools *do*, and how they can affect older adults’ and healthcare professionals’ practices.

### Background

Researchers use physical performance tests, observations, interviews/questionnaires, and rating scales to assess and document a baseline and changes in physical function and social participation, for example, Canadian Occupational Performance Measure (COPM; Law et al., 1998) and Barthel 20/100 (Mahoney & Barthel, 1965). They can also assess health-related quality of life, anxiety, and/or depression (Herdman et al., 2011; Wahlberg & Rose, 2015). Although assessments and documentation are valuable in clinical practice, Carrier and colleagues (2015) argue that standardisation of occupational therapists’ practice limits consideration of older adults’ actual wants, capacities, and needs. Consequently, standardised use of assessment packages may hamper occupational therapists’ professional discretion and practice. Interventions, such as reablement programs, may be designed to meet pre-set outcome measures, as opposed to documenting changes that are relevant for the individual in question. Timmermans and Epstein (2010) argue that promulgation and enforcement of standardised outcome measurements is a central form of social regulation, which is evident when older adults have to fit the program to be eligible.

Studies show that occupational therapists are generally positive towards the use of measuring tools, even though implementation of measuring tools can be problematic (Burton et al., 2013; Heiwe et al, 2013; Lindström & Bernhardsson, 2018; Pedersen & Kristensen, 2016; Sansonetti & Hoffmann, 2013; Upton et al., 2014). Occupational therapists are often caught in a dilemma between taking a person-centred approach and carrying out standardised practice as person-centred rehabilitation programs do not fit with standardised or mainstreamed practice guidelines (Carrier et al., 2015; Pedersen & Kristensen, 2016). Mandatory outcome measurements challenge the core value of person-tailored assessment and outcome evaluation, and they are a barrier to implementation of person-centred approaches (Pedersen & Kristensen, 2016).

There appear to be two strong drivers for the current trend of assessing, measuring, and uncovering capacities in older adults. First, there is a shift toward self-management and an institutional context of contractualisation of services in the welfare states aiming to save time and money (Carrier et al, 2015; Fletcher et al., 2019; Greve 2020). Second, the soaring evidence for the health benefits of physical activity across lifespan obliges healthcare professionals to focus on health literacy and facilitate health promoting activities (Bauman et al., 2016; Johnson et al., 2016). These trends lend support to a critical analysis of reablement practice development projects, both worldwide and in Denmark, exploring what measuring tools *do*, and how using measuring tools may empower or disempower the person in question.

### Theoretical Framework

This article applies a Foucauldian lens by use of his concepts of power and governmentality (Foucault, 1988; 1990; 1995), as used in several studies of occupational therapy (Laws, 2011; Mackey, 2007; Molke, 2009; Molke & Rudman, 2009; Rudman, 2005). Hammell (2013) argues that a Foucauldian appreciation of rehabilitation shows how this practice produces categories, assessments, measurements, and adjustments of individuals to make them fit in relation to a socially constructed and valued norm. A Foucauldian lens challenges the otherwise taken-for-granted aspects of occupational therapy practice, and it challenges researchers and practitioners to develop a better understanding of how power, as actions and practice, can operate through occupational therapy (Mackey, 2007; Molke & Rudman, 2009). Measurements delimit who older adults may become when under assessment; that they effect professionals’ actions and they effect activation policies and resource allocation (Nicholls et al., 2018). Foucault’s concept of power as productive and of governmentality as a conduct of actions directs our focus to what measuring tools *do*, apart from producing numerical scores. In this respect, these articles are based in a post-structural, French epistemological position (Beedholm et al., 2014; Kelly, 2019).

Foucault’s concept of governmentality describes how actions upon other actions conduct social practices, for example, how the action of measuring act upon reablement programs. The concept of governmentality captures the processes, tactics, and techniques of conduct of conduct of individuals or of groups, and the counter-conduct and critical attitude it produces (Foucault, 1988; 1990; Lorenzini, 2016). To “conduct” is at the same time to “lead” others and is a way of behaving within a more or less open field of possibilities. The exercise of power consists in guiding the possibility of conduct, that is, leading, and putting in order the possible outcome. The exercise of power is the conduct or leading of people’s conduct or behaviour. Foucault shows how social practices are situated and productive, and how they produce particular kinds of knowledge and truth about the matter of interest, for example, older adults’ eligibility for reablement. Power is everywhere, diffused and embodied in discourse, knowledge, and “regimes of truth,” and, therefore, power is neither agency nor structure, but is constituted by accepted forms of knowledge and is productive (Foucault, 1990). Governmentality captures the essence of self-management, that is, conducting people to conduct themselves (Foucault, 1995). The conduct of conduct can be described as a social technology to regulate social behaviour. Using Foucault’s concepts of power and governmentality as a lens on the use of measuring tools implies an appreciation of the capabilities the tools have to effect and be affected by the context in which they are used.

## Method

The article was based on a thematic analysis of empirical material consisting of reports from 13 different practice development projects on reablement programs for older adults in Danish municipalities. All projects stressed the importance of measuring tools to justify their local reablement models, even though they chose different tools. Denmark represents a typical north-European welfare state, and a critical analysis of these projects can add to the body of knowledge on reablement in Western welfare states (cf. Foucault, 1990).

### Strategy for Collecting Empirical Material

Empirical material was collected based on six inclusion criteria:

1. The project was publicly available on a municipality website.
2. The project was carried out from 2007 to 2012.
3. The project had a minimum of two web-published reports: a project plan and a final report.
4. The project problematised and aimed to change existing practice in municipal care for older adults in Denmark.
5. The project focused on changing the organisation of care services for older adults through a change in the involvement of the older adults by focusing on self-management, participation, involvement, and activity.
6. The project made use of measuring tools.

The search for practice development projects about reablement of older adults in municipal homecare began on the homepage of the Danish government’s funds for the development of better care for older adults (PDBCE) in 2007–2011. This homepage guided the search to concrete projects at municipal websites, in the areas where the projects originated. Many of the projects had applied unsuccessfully for financial support at PDBCE, had not been realised, and had thereby been removed from the municipal websites. By snowball technique, this search at the municipal websites led to other municipal reablement projects in the given time period. The authors screened the projects against the inclusion criteria for the present study, and their project plans and final reports were downloaded. All projects that met the inclusion criteria were included in the study. Twenty-two projects met criterias 1–5, and 13 projects met criterias 1–6. The empirical underpinning of the arguments in this article are based on the 13 projects that met all inclusion criteria.

### Analytic Strategy

Inspired by Braun and Clarke (2006) and Foucault’s concept of power and govermentality, a thematic analysis was conducted. A Foucauldian analytical lens directs attention towards social actions—that is, what people *do* and how they influence each other’s actions. The hidden and adverse effects of using measurement tools were analysed by asking questions such as who, what, where, which, why, how, and when to the empirical material. The empirical material was read in its entirety several times by both authors, first for an overview of content and context, and then a re-reading using Foucault’s concepts of governmentality and power as a lens (Foucault 1988, 1990). The critical analysis of the material uncovered how measuring tools and measurements were used to legitimise and justify allocation of resources, advice given, or treatment offered, and what effect this had on older adults and professionals. Conduct of conduct, counter-conduct, or critical attitudes were guiding concepts, and deliberately looked for in the entire material. This reading created sensitivity for inscribed rationalities in procedures and techniques, for example, detecting new aspects of use of measuring tools in reablement, which acted as both drivers and barriers to person-centred practice in occupational therapy. This analytical process had been a creative and dialectic process between the authors, keeping the theoretical framework tightly in mind and with the awareness that all measuring tools used in the included studies were also included in the analysis. Three themes were constructed: (a) Categorisation of loss; (b) Conduct of conduct: Self-tracking and competition; and (c) Conforming to expected forms of everyday living.

## Findings

Fourteen different measuring tools were identified in the 13 projects (see Table 1). The majority of tools are based on interviews of the older adults, and quantified qualitative assessment of occupations and tasks (Canadian Occupational Performance Measure [COPM], European Quality of Life–5 Dimensions [EQ-5D], Short Form [36] Health Survey [SF-36], Barthel 20/100, Visual Analogue Scale, Satisfaction on activity [VAS], and Assessment of Motor and Process Skills [AMPS]). The other measuring tools (Senior Fitness Test [SFT], Timed Up and Go Test [TUG], Sit-To-Stand [STS], Functional Independence Measure [FIM), Wii-Bodytest, and 2.45 Walking test) were scored by quantitative means as metre, seconds, or kilograms.

**Table 1:** Measuring Rools Used in the Projects

Measuring Tools	Measurements	Title of Project (Reference)^a^
Assessment of Motor and Process Skills (AMPS)	The performance quality of activities of daily living, based on direct skill evaluation while the person performs a specific task in dynamic interaction with the environment	Exercise before permanent help (Lyngby-Taarbæk Municipality, 2009)
Barthel-20	The ability to care for oneself, and the improvement of this ability (functional evaluation)	Active care (Faaborg-Midtfyn Municipality, 2010).
Barthel 100	The ability to care for oneself, and the improvement of this ability (functional evaluation)	Focus Shift from help to self-help and better level of functioning in the elderly. (Hoeje-Taastrup Municipality, 2009a; 2009b) Catch the everyday (Tonnesen et al, 2012).
Bodytest, incl. BMI or FIT age (Wii)	Body Mass Index Fit age calculated on background of age, sex, balance and SFT test	Wii in Trige (Aarhus Municipality, 2010)
Canadian Occupational Performance Measure (COPM)	The person’s self-perception of performance in everyday living, over time	Life-long activity (Copenhagen Municipality, 2010) Active care (Faaborg-Midtfyn Municipality, 2010). Maintain coping ability - active training (Jensen, 2010) Catch the everyday (Tonnesen et al, 2012). Lifestyle Redesign® i Aalborg Municipality (Loekken and Overgaard, 2009) Life-long living (Fredericia Municipality, 2015) Rehabilitation in Kerteminde Municipality (Kerteminde Municipality, 2007) Exercise before care (Billund Municipality, 2011) Exercise before permanent help (Lyngby-Taarbæk Municipality, 2009)
European Quality of Life – 5 Dimensions (EQ-5D)	Quality of life assessed by mobility, self-care, usual activities, pain/discomfort, and anxiety/depression	Active every day. Training in everyday life (Rudersdal Municipality, 2012). Focus Shift from help to self-help and better level of functioning in the elderly (Hoeje-Taastrup Municipality, 2009a; 2009b) Life-long living (Fredericia Municipality, 2015)
Functional Independence Measure (FIM)	A person’s level of disability as well as change in status in response to rehabilitation or medical intervention. Assessed by physical, psychological and social function	Early and targeted initiatives (Ringsted Municipality, 2010) Exercise before care (Billund Municipality, 2011)
Senior Fitness Test (SFT)	The underlying physical parameters associated with functional ability, identifiing whether an older adult may be at risk of loss of functional ability	Maintain coping ability - active training (Jensen, 2010) Early and targeted initiatives (Ringsted Municipality, 2010) Exercise before care (Billund Municipality, 2011) Wii in Trige (Aarhus Municipality, 2010)
Short Form (36) Health Survey (SF-36)	Health status assessed by vitality, physical functioning, bodily pain, general health perceptions, physical role functioning, emotional role functioning, social role functioning, and mental health	Lifestyle Redesign® i Aalborg Municipality (Loekken and Overgaard, 2009) Life-long living (Fredericia Municipality, 2015)
Sit-To-Stand (STS)	Functional lower extremity strength in older adults	Exercise before permanent help (Lyngby-Taarbæk Municipality, 2009)
Timed Up and Go Test (TUG)	A person’s mobility, including static and dynamic balance	Training prior to or in conjunction with assistive tools (Aalborg Municipality, 2011 L)
Visual Analogue Scale, Satisfaction on activity (VAS).	A person’s satisfaction with their level of everyday activities	Active every day. Training in everyday life (Rudersdal Municipality, 2012).
2.45 Walking Test	Basic mobility, understood as the ability to get up and sit down on a chair, walking over shorter distances and turning	Focus Shift - from help to self-help and better level of functioning in the elderly (Hoeje-Taastrup Municipality, 2009a; 2009b)

*Note*. Authors’ translation.

The detailed results of the reports of the 13 projects were based on actual project participants. Professionals’ pre-screening of eligible participants excluded adults who did not meet a minimum standard of “rehabilitation potential”. Occupational therapists participated in all 13 projects (see Table 2).

**Table 2:** Professionals Involved in the Projects

No	Title of Project^a^	Professionals Involved in the Project
1	Life-long activity (Copenhagen Municipality, 2010)	Occupational therapists and other employees at activity centers
2	Active every day. Training in everyday life (Rudersdal Municipality, 2012).	Occupational therapists and physiotherapists
3	Active care (Faaborg-Midtfyn Municipality, 2010).	Occupational therapists and physiotherapists
4	Maintain coping ability - active training (Jensen, 2010)	Occupational therapists, physiotherapists and nurse assistants
5	Focus Shift - from help to self-help and better level of functioning in the elderly. (Hoeje-Taastrup Municipality, 2009a; 2009b)	Occupational therapists, physiotherapists and other employees in the homecare service
6	Catch the everyday (Tonnesen et al, 2012).	Occupational therapists, physiotherapists, nurses, and other employees in the homecare service
6	Lifestyle Redesign® i Aalborg Municipality (Loekken and Overgaard, 2009)	Occupational therapists
7	Life-long living (Fredericia Municipality, 2015)	Occupational therapists, physiotherapists, nurses, and other employees in the homecare service
8	Rehabilitation in Kerteminde Municipality (Kerteminde Municipality, 2007)	Occupational therapists and other employees in the homecare service
9	Early and targeted initiatives (Ringsted Municipality, 2010)	Occupational therapists and other employees in the homecare service
10	Training prior to or in conjunction with assistive tools (Aalborg Municipality, 2011 L)	Occupational therapists, physiotherapists and other employees in the homecare service
11	Exercise before care (Billund Municipality, 2011)	Occupational therapists, physiotherapists and nurse assistants
12	Exercise before permanent help (Lyngby-Taarbæk Municipality, 2009)	Occupational therapists and physiotherapists
13	Wii in Trige (Aarhus Municipality, 2010)	Occupational therapists, physiotherapists and nurse assistants

^a^ Authors’ translation.

### Categorisation of Loss

Several measuring tools used in the projects provided rank order of a given matter in accordance with predefined hierarchies of potential outcomes. When it came to human function, the ranking order was accompanied by a “normal range” and definitions of scores below or above the normal range. For example, several projects used the *Barthel 20* or *Barthel 100* index to assess functional capacity in relation to activities of daily living (ADL) (Faaborg-Midtfyn Municipality, 2010; Hoeje-Taastrup Municipality, 2009a; 2009b; Tonnesen et al., 2012).

Barthel assesses the performance of the following 10 activities; eating, moving from bed to chair, personal hygiene, visiting the toilet, bathing, mobility, walking on stairs, dressing, bowel control and bladder control. The assessment is carried out by professionals of the interdisciplinary teams. (Tonnesen et al, 2012, p. 64/authors’ translation)

*Barthel* 20/100 were scored by a healthcare professional or by the older adult him/herself and detected potential shortcomings in self-care (Faaborg-Midtfyn Municipality, 2010; Hoeje-Taastrup Municipality, 2009a; 2009b; Tonnesen et al., 2012). The scores were ordered in five categories of dependency: total, severe, moderate, slight, or minimal. These categories might be useful for decision-making and resource allocation, but might also be at odds with the older adults’ experiences or self-presentation. The *Barthel* index was inscribed with a particular rationality, for example, separate self-managing persons from persons in need, or legitimate and illegitimate recipients of care. Even though the *Barthel* score was acknowledged as an eminent tool for a quick assessment of function, it could also assert pressure on everyday habits and preferences such as conduct of conduct or pressure of conformity.

Other projects used the *FIM* (Billund Municipality, 2011; Ringsted Municipality, 2010) and *SFT* (Aarhus Municipality, 2010; Billund Municipality, 2011; Jensen, 2010; Ringsted Municipality, 2010), which operated a similar categorisation of test persons. *STF* as an example categorised older adults into four groups:

SFT (Senior Fitness Test) is a physical test for older persons. It consists of six subtests that test the strength, endurance, agility and functional mobility of older persons. Each partial result says something about the participant’s level of functioning in relation to the participant’s biological age. There are four categories:

A. Risk of losing functional mobility

B. Below average

C. Normal

D. Above average. (Jensen, 2010: p. 5/authors’ translation)

The category “normal” represented the average and expected functional ability in relation to chronological age, where the outcome measurements represented biological age, defined as a measure of how the body carried inner and outer signs of ageing (Aarhus Municipality, 2010; Billund Municipality, 2011; Jensen, 2010; Ringsted Municipality, 2010). *SFT* departs from a rationality where ageing and loss of function were parallel processes. The “normal” category was based on a statistical “normal” describing of how function ought to be at a certain age. The test was a technique and a procedure that could be used to identify older adults’ eligible for reablement services or in need of preventive interventions. As *Barthel* index, the tools carried along ideas about self-sufficiency as a normative golden standard, which did not take into consideration the collective nature of human health and everyday living, or the different values with which older adults themselves imbued their mental, social, or functional capacity. The outcome measurements legitimised possible interventions and acted as a social technology to observe, collect, and compare older adults’ capacities. This knowledge was value-laden, that is, the best outcome was a lower fitness age than chronological age. Such social technologies served as a way to regulate and optimise social behaviour by encouraging older adults to take action and increase their physical functions, and thereby achieve a younger “biological age” in the next measurement. The categories did not focus on the intersection between capability, rest-function, and contextual resources, but on evident or expected loss. Measuring, scoring, and categorisation accordingly effected and directed attention to needs instead of wants, and loss instead of capacity. This was useful for some purposes (e.g. prioritisation for reablement), but could be detrimental for an older person’s experiences, self-presentation, and/or hopes for the future if they were less fit than average for her/his age group.

The *SF-36* (Fredericia Municipality, 2015; Loekken and Overgaard, 2009) and *EQ-5D* (Hoeje-Taastrup Municipality 2009b), patient-reported measurement of generic health status, were also used in some projects. The *EQ-5D* can serve as an example:

The test itself consists of two parts. The first part is an index with five questions/dimensions. The questions concern movement, personal care, usual activities, pain/discomfort and anxiety/depression. […] possible answers: no problem (score 1), some problems/moderate (score 2) or cannot/extreme problems (score 3). The five questions together create a health profile that can be translated into an overall number […] between 0 and 1, where 0 = death and 1 = perfect health state. (Hoeje-Taastrup Municipality, 2009a: p.20/authors’ translation)

The *EQ-5D* questionnaire was formulated in an “I-style,” for example, “I have moderate problems in walking about” or “I am unable to walk about” (Hoeje-Taastrup Municipality 2009b). The questionnaire aimed to detect and score decline and loss, and had a double edge: categorisation and conduct of conduct. When persons who experienced minor, moderate, or major challenges in everyday living were subjected to a test, they were lectured about their responsibility to keep fit and be self-sufficient. The test results were not directed towards capabilities, resources, and wants. Accordingly, the rationality of ageing represented in the test results did not capture the duality of ageing in the form of oscillations between possibilities and loss; it only captured and categorised loss.

### Conduct of Conduct: Self-Tracking and Competition

Several projects used the *SFT*, *Barthel 20/100* and *COMP* (Aarhus Municipality, 2010; Billund Municipality, 2011; Copenhagen Municipality, 2010; Faaborg-Midtfyn Municipality, 2010; Fredericia Municipality, 2015; Hoeje-Taastrup Municipality, 2009a; 2009b; Jensen, 2010; Loekken and Overgaard, 2009; Ringsted Municipality, 2010; Tonnesen et al, 2012). These tools were designed to assess and document deviations from a predefined standard of normality, and they carried a social appreciation of healthy ageing and optimal functioning. It could be regarded as conduct of conduct by design. However, this could also be interpreted as a demonstration of potential capacities and functions, that is, bettering of function was possible and a better score could be assigned. As such, the tests had built-in motivational or competitive potential (e.g. Billund Municipallty, 2011; Hoeje Taastrup Municipality, 2009a; Kerteminde Municipality, 2007; Loekken and Overgaard, 2009). Some older adults detected personal strengths and possibilities when fulfilling the tests:

The patient becomes aware of his own potential for development despite any health difficulties. […] Where COPM was used, the patient had an increased quality of life, as the patient’s own assessment of skill level had changed during the training process. The patient finds s/he is able to perform activities that were previously problematic. (Kerteminde Municipality, 2007: pp. 14-15/authors’ translation)

For those who are told that they are doing well in the test and above average, it was a motivation to remain active. For a participant who got a bad result, it could be a motivation to become more active. (Hoeje-Taastrup Municipality, 2009a: p. 48/authors’ translation)

In addition, *VAS* was used to measure older people’s satisfaction with their level of activity following everyday rehabilitation (Rudersdal Municipality, 2012). The use of measuring tools and outcome measurements under this system could produce external motivation for social and physical activity, and self-care for older adults. Filling in a questionnaire, or answering oral questions, presupposed that the person honestly disclosed their health status and health-related behaviour. This disclosure had the potential to facilitate responsibility and served as a motivator for the patient to track their own progress and take action to better their own health. Measurement results might strengthen and empower older adults by providing evidence for their potential to better their health. However, participants without a “development potential” were previously excluded from the projects:

During the project period, the target group is defined as […]: All new clients with a rehabilitation potential […] Rehabilitation potential is defined as: development potential on one or more of the significant limitations in functional ability. (Tonnesen et al, 2010, p.30/authors’ translation)

Whether the result was good (above average) or bad (below average), it could encourage the test-persons to keep up healthy habits or pick up new healthy habits. When a test was scored, the results were compared with pre-set reference values created by aggregating results from previously tested persons. These persons were not present, but still served as competitors, and had an impact on the use of measurement. The 2.45 walking test, used in one of the projects (Hoeje-Taastrup Municipality, 2009a; 2009b), illustrated this:

The walking test is designed for frail older persons (65+), but is generally used for people with varying degrees of disability and cognitive difficulties. […] The 2.45 Walking Test [.] is an easy and simple test for older persons to understand and execute. The test does not require much space and therefore can be carried out in small homes. [….] it effectively reflects the development of the individual in relation to the walking function, balance and fall risk. (Hoeje-Taastrup Municipality, 2009a: p. 20/authors’ translation)

Walking speed was regarded as a vital sign. The test’s rationality was the correlation between walking speed and overall health status. Speed was modifiable, and was an eminent example of a measurement that conveyed important information to professionals and to motivated older adults. It was a test with great potential for “conduct of conduct” of an older adult and for motivating both older adults and healthcare professionals, but in different respects.

### Conforming to Expected forms of Everyday Living

All measuring tools had per se a corresponding set of reference values and differentiated between older adults with and without a particular (functional) problem, for example, walking measured by 2.45 Walking Test (Hoeje-Taastrup Municipality, 2009a), mobility measured by TUG (Aalborg Municipality, 2011), functional strength measures by STS, and performance quality of ADL measured by AMPS (Lyngby-Taarbæk Municipality, 2009). When a measuring tool was used, stakeholders might be ignorant about the fact that reference values were created for populations, not individuals, and mean values did not capture normal variations on an individual level. Measurements that produced numbers were regarded as indisputable facts, whereas measurements that produced categories might be referred to as exercising good or bad professional discretion. The calculation of body mass index (BMI) used in one of the projects (Aarhus Municipality, 2010) might serve as an example. As with walking, a *BMI* score did not tell the whole story and might effect the person in question negatively.

I have always been overweight. I feel the test has touched a nerve in me, it tells me the result and I really do not like it. But on the other hand, it may give one a kick [Interview with an older adult]. (Aarhus Municipality, 2010, p.14/authors’ translation)

In addition, during an observation study as a part of the project (Aarhus Municipality, 2010), it was noted that an older adult apparently did not like to be invited to lose weight, and the observer reflected subsequently:

We have talked before about using the Wii, which can be a motivating factor in training. However, it can also be a disincentive to start being reminded that you are overweight [observer reflection]. (Aarhus Municipality, 2010: p.14/authors’ translation)

*BMI* and walking speed provide quick facts about health and function. A correlation between walking speed and general function is well known as a correlation between health risks and being overweight. The reference values on body weight and physical activity were apprehended as causality and an expert assessment for any individual, which justified the introduction of Wii, which is exer-gaming (Aarhus Municipality, 2010). At the same time, use of *BMI* functioned as both a diagnostician and a health director. Comparing and reporting individual results in relation to reference values meant that professionals could present themselves as ignorant messengers, and through the measurement, advise the older adults to change their lifestyle and habits. In that way, use of measuring tools and outcome measurement functioned as social technology, having an inherent power to regulate and rule behaviour of older adults by promoting conduct of conduct, and exercised a pressure towards conformity. The measuring tools acted as an “independent” or “external” expert, who could leave the relationship between older adults and healthcare professionals untouched. In the excerpt from the Aarhus report earlier, causality between sedentary lifestyle and being overweight was implied by the BMI measurement. The quotes showed three different reactions towards the authoritative advice in the BMI-number: motivation, counter-conduct or a critical attitude, and second thoughts about the advice given. The example showed that use of measuring tools was accepted as a health expert assessment, and facilitated motivation and resistance, but also prompted critical reflection and discretion in professionals.

## Discussion

The discussion will focus on three main findings of hidden and adverse effects of using measuring tools in occupational therapy, namely, that measuring tools (a) represented older adults in pre-defined, administrative categories; (b) influenced the conduct of conduct, and )c) extended radius of action for professional.

First, the findings show how measuring tools categorised older adults in predefined categories according to their losses and worked as a social technology tended to produce generalised truths about older adults and predicted outcomes of reablement. The findings show that “biological age” was predominant in representing older adults and their everyday function. The backdrop for this discussion is the global trends of longevity, prevention of non-communicable diseases and policies on active ageing (European Commission, 2012; WHO, 2002). The World Health Organization (WHO) appointed 2012 as the European Year of Active Ageing and Solidarity between Generations (European Commission, 2012). In recent years, active ageing has become both a framework for understanding healthy ageing and a key concept for both the EU and WHO (Lassen, 2014; WHO, 2002). Understanding of active ageing focuses on ageing as a process that is shaped over a life cycle where lifestyle factors, including one’s activity level, are important. The current study shows that the use of measuring tools does not take into consideration older adults’ own understandings of active ageing, such as being able to drink and play pool in an activity club or enjoy knitting and coffee with friends (Lassen, 2014). Van Dyk and colleagues (2013) show that lifestyle choices allow older adults to keep their ailments “at arm’s length” or as a valued part of their ageless identity. Further, van Dyk and colleagues (2013) show that older adults resist activation policies, as they frame ageing as a continuum of life into older years. The use of measuring tools in current study points to what Nicholls and colleagues (2018) warn against, namely an instrumentalisation of activities and a medicalisation of everyday living. The current study shows that measuring tools are not able to pay heed to different socio-cultural and economic differences between individuals or cohorts of age-groups as such. All these categories may serve certain aims, including identifying those eligible for reablement, Regardless, measuring tools still hold the potential to obscure the focus on capacities for change and improvement of the tested person. An uncritical use of measuring tools risks the results being rejected or circumvented by counter-conduct and critical attitudes by those they are meant to serve, both healthcare professionals and their clients. Conversely, in line with Foucault, resistance to measuring tools can also lead to a space for change of practice.

Second, the findings show that measuring tools also were used for assessment, self-tracking, motivation and influenced the conduct of conduct. The use of measuring tools in the practice development projects are clearly connected to health promotion and prevention, and identification and elimination of health risks (Lupton, 2014). Modern health prevention is above all the tracking down of risks, where risk is understood as a composition of factors that make a risk probable (Rabinow, 1996). This means that prevention becomes surveillance of likely occurrences of diseases, abnormalities, and deviant behaviour to be minimised, *and* (promotion of) healthy behaviour to be maximised. The current findings repudiate the idea that interaction between older adults living at home and healthcare professionals can be framed as a one-way delivery of services. However, homecare and reablement are co-created and co-produced through the practice development projects. The measurement outcomes thus have the capacity to track, motivate, or provoke older adults and healthcare professionals, and facilitate discussions and clinical discretion. Through interactions with older adults, professionals have an opportunity to ameliorate the effects of being described as “disabled,” “dependent,” or “lacking” according to pre-set standards. The use of measuring tools in current study categorised and represented the older adults through standardised measurements and created a particular matter of concern, which demanded healthcare actions. Use of measuring tools had an intrinsic power to transform pleasure and natural movement in everyday life into a logic of medicine with a need for lifestyle change, which served to maintain the vital functions of the body (Nicholls et al, 2018; Pedersen and Saltin, 2015).

Forthcoming implementation of the project interventions and use of measuring tools into daily working practice opens up possibilities to control both healthcare professionals’ actions and the older adults who are considered worthy of professional attention. Thus, Pedersen and Kristensen (2016) show how occupational therapists argued that a standardised measurement was restricted to a focus on specific issues in functioning, and consequently, other important issues were left out. In addition, use of measuring tools can act as a quality assurance instrument, where it is possible to compare and price healthcare actions carried out in relation to older adults. The current findings show a unanimous tendency to mainstream the homecare services. This is often bound to tight economic priorities in the form of resource allocation, priority settings and cost-effectiveness, in contrast to the contemporary appreciation of person-tailored care (Bulamu et al., 2015; Lewin et al., 2013). The current study shows how participants without a “development potential” were previously excluded from the reablement projects. Inspired by Alvsvåg’s (1985) terminology, it means that the “ill ill” persons are not offered rehabilitation efforts as they are not worth the investment—neither from an economic perspective, a project success criterion perspective or a human perspective.

Third, the findings show that measuring tools created an extended radius of action for healthcare professionals’ authority. Although measuring tools primarily produce a representation of the tested person, healthcare professionals get access and opportunity to intervene in the tested persons’ life and body. Andersen (2003) described this as to “hack” a person, to impose an unwanted or unknown change, where use of measuring tools can be considered as a social technology, which extends the professional’s impact area and, at the same time, constrains critical discretion and evaluation of individual relevance. Self-technology is characterised as a way to make a human being a subject (Foucault, 1988). In this regard, a technology does not need to be a materiality but can also be something imaginary, such as a measuring tool that encourages thinking and acting in certain ways. Gardner and Cribb (2016) show how *COPM* structures activities of individuals in clinical settings, and how it is implicated in initiating and supporting particular modes of patient involvement, and thereby COPM actively structures power relations and is engaged in forms of ethical work, which may or may not be desirable by clients, professionals, and/or the society. They show how COPM represents a form of “power storage,” when healthcare professionals adopt the tool into their clinical practice where the power relations it prescribes are enacted endlessly and thus become institutionally embedded (Gardner and Cribb, 2016). This can be regarded as an institutionalisation of patient-centred tools in the form of techno-governance (May et al., 2006). In that way, use of measurement tools govern the tensions in clinical settings of the potentially conflicting ideas of evidence-based occupational therapy and patient-centred occupational therapy (Gardner ad Cribb, 2016). According to May et al. (2016), and technogovernance entails the prescribing of clinical practices by which patients are guided and their narratives are appropriately situated. Gardner and Cribb (2016) argue that patient-centred interactions entail micro-power dynamics that are asymmetrically distributed and considerably more complex than the more egalitarian models often mobilised by advocates of patient-centeredness. Reference values emerge as indisputable facts, also in patient-centred occupational therapy, and thus tend to act in an authoritarian and paternalistic manner (Gardner and Cribb, 2016). The result of the measurement is a generic representation of a particular subject matter, for example, BMI, which is designed to minimise impact of characteristics other than the measured factor such as the relationship between body weight and height. Measurements are part of assessments, and uncover lacks, losses, or rest-capacities, which might affect encounters between older adults and healthcare professionals (cf. Foucault, 1990). Practice development projects, use of measuring tools, use of outcome measurements, and the intention of the forthcoming implementation in clinical practice could also be regarded as an attempt towards professionalisation of occupational therapy. Despite an ideology about client-centred practice in OT, the medical profession has influenced occupational therapy, in particular by positivistic thinking and reductionist-oriented practices (Hammell, 2013; Pierce, 2014). The simultaneous emphasis on measurement outcomes in numbers, causality, and reductionism in occupational therapy contextualises ranges of diverging values and could be regarded in close connection to the logic of evidence-based practice (EBP) and rationality in the profession of medicine (Heiwe et al, 2011). In the process of professionalisation of occupational therapy, the ideal of EBP currently plays a pivotal role in consolidating occupational therapy as an independent profession. At the same time, occupational therapy maintains its subordinate position to the medical profession through mirroring on the same understanding of science as medicine par excellence (Heiwe et al., 2011; Lindström & Bernhardsson, 2018; Upton et al., 2014).

Finally, the present study has its limitations. The search for rehabilitation projects in Danish municipalities has captured many existing projects. However, it is not possible to know if the snowball sampling has led to the total number of projects. Regardless, all these projects represent the use of measuring tools that still are current and relevant. Whether several included studies would have shown that the analysis could be supplemented with additional patterns must remain unknown. The empirical material is produced from reports of completed practice development projects. We do not have first-hand data or access to the material on which the reports were based, which means we have analysed the text of the written reports, and have not analysed the empirical material on which these reports were based. Using Foucault’s theoretical framework as an analytical lens made it possible to minimise the preconceptions of the researchers. It is per se an impossibility to explain one’s pre-understandings. However, by moving one’s pre-understandings to a theoretical perspective, it is possible to be stringent and transparent in the analysis with limited disruption of the researchers’ own, unconscious pre-understandings, which strengthen the study’s trustworthiness. According to Foucault (1990), in Western societies, the power structure and logic of medicine as a web of underlying understandings and thought patterns are everywhere and omnipresent. This means that the findings of this study probably also could be found in other countries, in use of measuring tools in other healthcare professions’ practice development projects, and in use of measuring tools in occupational therapists’ daily clinical practice.

## Conclusion

This article illuminated hidden and adverse effects of using measuring tools in occupational therapy. The measurements provided quick facts about functioning, and it assigned the tested persons to pre-designed categories to inform professionals about needs or self-reliance. Simultaneously, outcome measurements imposed an understanding of health and functioning upon the older adults that might contradict their own understanding of their general health. As social technologies, use of measuring tools were powerful in producing generalised truths about being an older adult. Use of measuring tools effected the individual’s self-perception and understanding of normality, which could direct individuals to take specific actions—and/or refrain from others. Further, use of measuring tools produced motivation for both older adults and professionals to take action on selected issues, and outcome measurements emerged as an assertion from a health expert. The use of measuring tools also functioned as an extension of healthcare professionals’ authority and had the potential to empower or disempower older adults and occupational therapists. Overall, use of measuring tools can be regarded as attempts to professionalise occupational therapy, whilst simultaneously maintaining occupational therapy in a subordinate position to medicine.

This study calls for further research on the social and relational complexity of use of measuring tools where both the healthcare professionals and older adults’ practices and perspectives on use of measuring tools are taken into account.

### Key Messages

- Occupational therapists must take into consideration that use of measuring tools and outcome measurements have hidden and adverse effects, which can empower and disempower older adults and occupational therapists.
- Uncovering the way in which use of measuring tools are implicated in structuring and managing power relations can help occupational therapists to reflect upon their profession.
